# Understanding how to create healthier places: A qualitative study exploring the complex system of urban development decision-making

**DOI:** 10.1016/j.healthplace.2023.103023

**Published:** 2023-04-18

**Authors:** Anna Le Gouais, Geoff Bates, Rosalie Callway, Heeseo Rain Kwon, Lisa Montel, Sian Peake-Jones, Jo White, Md Nazmul Hasan, Caglar Koksal, Andrew Barnfield, Krista Bondy, Sarah Ayres

**Affiliations:** a University of Bristol, Bristol Medical School (Population Health Sciences), UK; b University of Bath, Institute for Policy Research, UK; c University of Reading, Henley Business School, UK; d University of Bristol, Law School, Centre for Health, Law and Society, UK; e University of Manchester, Manchester Urban Institute, UK; f University of the West of England, Centre for Public Health and Wellbeing, UK; g University of Bath, School of Management, UK; h University of Bristol, School for Policy Studies, UK

**Keywords:** Urban development, Decision-making, System, Qualitative, Environmental determinants, Population health

## Abstract

Tackling complex system challenges like creating healthy environments requires understanding priorities and structures affecting multiple actors. This qualitative study, involving 132 multi-sectoral stakeholders spanning the urban development decision-making system, explores how to influence healthier place-making. Using thematic analysis we develop themes around competing stakeholder priorities; structural ‘rules’ and influential relationships; and justifying a focus on health, requiring greater clarity and consensus around definitions of ‘healthy’ urban development. Building on the socio-ecological model we highlight how a multi-faceted approach is required for change at multiple levels in the complex system to target individual actor motivations, organisational priorities and structural ‘rules’.

## 1 Introduction

The urban environment can influence population health due to associated risk of non-communicable disease. For example, housing density, transport infrastructure, and accessibility of amenities, services and employment will affect travel behaviours, levels of physical activity and air quality, with associated health impacts (e.g. risk of cardiovascular disease, diabetes, stroke, cancers, mental ill-health and premature mortality) (Samitz et al., 2011; Newby et al., 2015; Cavill et al., 2019; Ortiz et al., 2022). Green infrastructure, including parks and trees, affects mental health through impact on social cohesion, physical activity and environmental factors including air quality and visual stimulation (Sandifer et al., 2015; WHO Regional Office for Europe, 2016; Chen et al., 2021), with potential for reducing inequalities (Browning et al., 2022). Economic costs of unhealthy environments are high, with poor air quality alone estimated to cost the UK over £20 billion annually (Royal College of Physicians, 2016). Climate change impacts associated with materials and embedded carbon from construction also impacts on planetary health with associated public health impacts (Bélanger et al., 2015; Rocque et al., 2021). Therefore urban development decisions, including for new urban extensions, inner city regeneration, creation of new towns, and changes to transport networks, can influence population health. These wider determinants of health involve many aspects of place-making, as outlined in Public Health England’s Spatial Planning for Health guidance (Public Health England, 2017) and the London Healthy Urban Development Unit’s Health Impact Assessment tool (NHS London Healthy Urban Development Unit (HUDU), 2019).

Urban environments can influence health outcomes in many ways and decisions that shape these environments are spread across many diverse stakeholder groups with differing and competing motivations and objectives (Blackman, 2006; Finka and Kluvánková, 2015; Holmes et al., 2017). Stakeholders span public, private and third sector organisations, as well as members of the public, with the latter directly experiencing the health impacts of development decisions. Dominant actors in the system are from non-health sectors including finance, property development and transport (Healey, 1991; Tasan-Kok et al., 2019).

Several systemic and/or structural barriers have been identified that prevent health from being prioritised in urban development decision-making in different contexts (Ige-Elegbede et al., 2020; Pineo et al., 2020; Black et al., 2021). These include, but are not limited to, siloed thinking in national and local governments; lack of coordination between public health professionals and other stakeholders; failure to leverage health evidence in key decisions; lack of power, capacity, and resources within public sector organisations; dominance of private companies leading to short-term thinking; and competing priorities leading to subordination of health to other non-health issues (Allender et al., 2009; Lake et al., 2017; Black et al., 2018, 2021; Fischer et al., 2018; Carmichael et al., 2019; Keeble et al., 2021).

We conceive urban development therefore as a complex network of stakeholders and processes which requires a systems lens to explore decision-making and its contributions to population health outcomes. Health research based on systems approaches attempts to improve public health by changing the complex systems that determine health outcomes (Rutter et al., 2017). As Diez Roux states: “from a systems perspective, health is conceptualised as an emergent property of a system, in which processes operating at the levels of individuals and populations are inextricably connected” (Diez Roux, 2011, p.1). A complex system represents the interactions and dynamics between a range of interrelated components. To truly understand decision-making in one area – and how it might ultimately influence health outcomes - we need to consider how that part of the system affects, and is affected by, multiple other parts of the system (Hawe et al., 2009). Similarly, considering urban development as a complex system is to understand that decisions made in one part of the system can have implications, intended or unintended, on many other parts of the system.

Applying a systems lens therefore supports understanding of how and why decisions are made, how the system works, and what interventions may be required for positive change. This represents a shift away from linear models whereby interventions are conceived as direct solutions to problems, and instead encourages consideration of interventions as ‘events’ in a complex system, seeking to disrupt a system that contributes to unwanted outcomes (Hawe et al., 2009; Moore et al., 2019). Multiple events that seek to change different points in the system can over time drive meaningful changes (Rutter et al., 2017). However, it is argued that a failure to understand how a complex system works, or over-simplifying problems, can lead to the implementation of ineffective or harmful interventions (Sterman, 2006; Atkinson et al., 2015).

Applying a systems perspective to studying decision-making in urban development encourages consideration of the role of factors beyond individual preferences, values, and beliefs. Organisations and institutions are also important, and governments are increasingly facing what are referred as ‘tangled’ problems (Dawes et al., 2009) involving a jumble of actors, goals and resources (Gasco-Hernandez et al., 2022). The socio-ecological model (Bronfenbrenner, 1978) has long been used to understand differing levels of influence on public health issues, looking beyond individual conceptualisations of decision-making and considering various important social, cultural and structural influences as a way to develop multi-faceted solutions. It has been used to understand ways to tackle various complex public health challenges, such as for active travel (Irwin et al., 2021), mental wellbeing and neighbourhood environments (Lauwers et al., 2021), community engagement (Caperon et al., 2022) and climate change (Pan et al., 2019). Reflecting that decisions are influenced by many factors across the system, the model suggests that interventions are needed that target different parts of the system if they are to bring about sustained change (Sallis and Owen, 2015), e.g. individual actors, to advocate for healthier development; organisations, to change company priorities to include health; and at structural levels, to influence policy frameworks or increase legal obligations to demonstrate health impact of decisions. Therefore we consider the socio-ecological model as a useful framework through which to consider complexity in the urban development system and how decision-making is made that shapes the design and creation of urban environment.

The impacts of unhealthy development are a growing concern: in an increasingly urbanising world, development decisions affect growing numbers of people, for both existing and new communities (United Nations Department of Economic and Social Affairs, 2019). Although there are examples of environmental changes that support health and wellbeing, including ‘15 minute neighbourhoods’ and Low Traffic Neighbourhoods (Aldred and Goodman, 2020; Laverty et al., 2021) many new urban developments are being built that do not enable healthy behaviours, nor support planetary health (Black et al., 2021; Spencer and Pendlebury, 2021). Car-dependent neighbourhoods with minimal opportunities for active travel and lack of green spaces and trees remain common outcomes of decision-making (Transport for New Homes, 2022).

Recent studies in the UK and internationally have explored some of the challenges of creating healthy places, focusing on particular parts of the urban development system, such as planning applications (Keeble et al., 2019; Netherton and Chang, 2020; Bicquelet-Lock, 2021) and post-planning approvals (UWE Bristol, 2021), or on limited features of healthier environments, such as active living infrastructure (Le Gouais et al., 2020a, b). However, there is a gap in understanding the health considerations across the broad, complex system of urban development decision-making across a wide range of stakeholders. This study seeks to address this, extending understanding about the role of health and actors in the system to provide insight into pathways to healthier place-making. This includes financial actors involved in funding new healthy urban development, which have been less explored, but are important, especially with limited local government resources following years of austerity (Harris et al., 2019; Le Gouais et al., 2020a). Clearer understanding of how health impacts are taken into account in decision-making across this system of actors and institutions is vital for identifying leverage points to promote the creation of healthier environments for population-level health and wellbeing. Therefore in this study we sought to understand what influences decision-making for individual actors, organisations, and structures in a complex system, addressing the research question: ‘How can the system of urban development decision-making be influenced to create healthier environments?’ This includes considering the priorities of different types of actors involved in urban development decision-making, particularly how health fits amongst other non-health priorities. This understanding was sought to support design of interventions to influence urban development decision-making for healthier environments to reduce risks of non-communicable diseases and address health inequalities, as part of a large research project: TRUUD (‘Tackling the Root causes Upstream of Unhealthy Urban Development’).

## 2 Methods

### 2.1 Participants

The explorative nature of our study meant we sought to include a wide range of stakeholders across the urban development system. To support this our team was comprised of researchers from a variety of academic disciplines, and with experience of working in public, private and third sector organisations. The team’s background spanned urban development, transport, public health, real estate, management, public policy, law and public involvement.

Purposive sampling was conducted, based on the inclusion criteria of stakeholder influence and expertise in urban development decision-making in England. Through desk-based searches, literature review, stakeholder mapping and a related pilot project (Black et al., 2021), a database of approximately 500 actors was generated, from which the interview sub-teams selected participants. Snowball sampling was also conducted (Bazeley, 2013). Participants were invited by email, with information about the purpose of the study: to understand factors influencing decision-making for healthy urban development. In total 123 semi-structured interviews were conducted with 132 interviewees across seven researcher sub-teams. Sub-teams tended to consist of two researchers from similar disciplinary backgrounds (one sub-team had only one researcher). Each focused on a group of stakeholders that aligned with their area of expertise, such as local government officers, local government elected members, national government actors, real estate actors, property developers and consultants. The general types of stakeholder are shown in Table 1, although these are broad categorisations of interviewee characteristics and actors may play multiple roles in the system with diverse combinations of expertise (Healey, 1991).

### 2.2 Data collection

Semi-structured interviews were conducted online due to Covid-19 restrictions between May and October 2021. They enabled flexibility and exploration of issues that were not identified a priori. Each sub-team developed interview guides for their specific stakeholder group. These covered: actors, institutions and networks; world views and perceptions of why healthy places are not created; and how decisions are made within the urban development system, including processes, resources and use of evidence. Questions about the role of community involvement, health economic valuation and legal considerations were also included.

All interviewees provided informed consent. Interviews were audio recorded and transcribed verbatim and took an average of 55 min (range 26–112). Ethical approval was obtained by the University of Bristol, reference 94162.

### 2.3 Analysis

We coded using deductive and inductive processes (Bazeley, 2013). Initial deductive codes were identified from literature review work spanning complex systems and actor-structure perspectives in the subject area of health and urban development decision-making (Hasan et al., forthcoming). These deductive codes were grouped into categories. Inductive coding was also used to provide flexibility for unanticipated issues identified in the data.

Members of each interview sub-team coded their own interview transcripts in NVivo 12 (QSR International Pty Ltd., 2018). In addition, one researcher who did not conduct interviews developed codes in relation to legal considerations across all interviews. Weekly team meetings were conducted where researchers could propose new inductive codes. These were discussed and new versions of the shared NVivo file created for all sub-teams to code to. The coding categories, used to group codes in NVivo, were also edited and added to during the coding process.

**Fig. 1:**
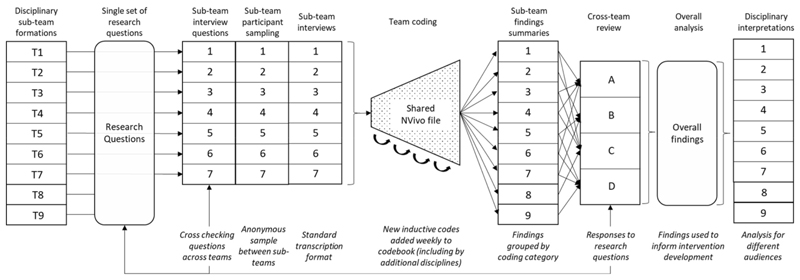
Stages for team coding and analysis of interview data (Bates et al., 2023).

After coding each sub-team summarised their own interview data to capture their own disciplinary findings, grouping these by the categories. They also summarised how their data responded to the interview topics. Two researchers from law and public engagement disciplines, who did not conduct interviews, produced their own disciplinary summaries using relevant codes covering the whole dataset. This approach enabled a form of ‘codebook’ thematic analysis, facilitating production of ‘domain’ summaries, while recognising some subjectivity by the researcher (Braun et al., 2018). Fig. 1 provides an overview of the team interview coding and analysis process.

The sub-team summaries, which ranged from 8000 to 58,000 words (including quotes), were read by the lead author (ALG). An interpretivist approach was conducted by ALG to compare findings from the separate summaries using a form of reflexive thematic analysis (Bazeley, 2013; Braun and Clarke, 2019) or thematic synthesis (Barnett-Page and Thomas, 2009). This enabled development of themes from across the broad, disciplinary-diverse data set to provide deeper insights than simply summarising the data. This was informed by meta-ethnography (Noblit and Hare, 1988), an approach originally developed for qualitative evidence synthesis to translate findings from multiple qualitative studies into each other. Stages of analysis included identifying and grouping key findings from each sub-team’s summary to support comparisons across the whole dataset, with mind maps developed to consider inter-related issues. This led to the development of higher-order, overarching themes (described in the Findings section), followed by further analysis to develop ideas that seek resonance (Tracy, 2010) (described in the Discussion section). This involved holistic analysis of the urban developed system, incorporating the views and experiences of a wide variety of actors.

## 3 Findings

We identified three main themes from the data which are relevant to the complex system of urban development decision-making and intervention development to influence the creation of healthier environments: Competing priorities; ‘Rules’ and relationships; and Justifying a focus on health. These are described below.

### 3.1 Competing priorities

A diversity of actors are involved in urban development decision-making. We identified competing priorities amongst three key groups: national government (including politicians, civil servants and government agencies); local/regional government (including councillors, officers, and regional bodies); and private sector for property development (including developers, investors, landowners and brokers). Their dominant values and intentions may result in either aligned or conflicting priorities which can influence health outcomes.

#### 3.1.1 National government priorities

National government actors had multiple, diverse priorities spanning housing, economy, environment, public opinion and political ideology. Trade-offs that affect health considerations relating to urban development appeared inevitable.

House building was described as a national government priority, with an emphasis on quantity of new homes, rather than quality. There was evidence of tension between layers of government associated with housing targets and belief that national government had chosen to retain significant control over local government, including over funding to achieve objectives.

“[local government and communities] don’t have enough powers and successive governments have talked about the planning agenda in different forms, but they’ve never really let go” (KR-494, national level politician)

National government priorities were described in relation to each separate department, which may compete. Support from Treasury, Cabinet Office and the Prime Minister’s Office were described as crucial for other departments to achieve their objectives. Other increasingly influential cross-cutting agendas included net zero carbon, climate change, biodiversity, levelling up, placemaking and beauty. These were said to create opportunities for related policies that directly support health objectives.

“… the environmental imperatives and the links to air quality and some of the aspects around clean green travel would be very helpful for health objectives. I don’t know if we think they’re at the top of the agenda but certainly it feels like there’s a real currency of those environmental issues which is really helpful to some of those agendas” (DB-243, Civil servant, health & urban planning)

There was broad agreement that the UK had a dominant car culture, although there were reports of “institutional change” in the Department for Transport (TC-230, Civil servant, transport) where cycling and walking had become more valued, with recognition that they were “relatively cheap … relatively impactful” (JH-388, national level public sector, property finance). Interviewees thought changing public opinion relating to climate change and public health concerns following Covid-19 may have influenced this, due to increased focus on sense of place and communities.

Conservative, libertarian ideology was seen as influential in the housing market, where home ownership was promoted over renting, and government had a limited role in providing social housing or funding place-making infrastructure. There was a view that “we need very brave politicians” (AB-250, Scientific advisor) to support healthy development.

“… there are a lot of people within the broader centre-right who, instinctively, resist the idea that government should be banning lots of things, telling people how to live their lives” (PR-234, national third sector organisation, urban planning)

#### 3.1.2 Local/regional government priorities

Local/regional government also had multiple, often competing, demands that influenced decisions associated with healthy urban development. These could result in trade-offs involving quantity and quality of housing, social needs, financial demands, public opinion and political concerns.

Local government was described as having dual roles to both facilitate and control development – to meet local housing need and national government housing targets, while also negotiating with developers for social benefits.

“… there’s a tension there in terms of as a local authority you want to be supportive of development … but often in practice those do come at a cost and the more constraints you put on development, sometimes the harder it is to get things developed, … there’s a balance between what you would maybe want to do in an ideal world and what you can do whilst still allowing development to progress.” (RK-308, local government, property development)

Financial limitations were discussed as barriers to achieving all desired development outcomes by politicians and officers – it appeared that local government officers had too many issues deemed to be priorities by leaders, without adequate acknowledgement of necessary trade-offs, e.g. commercial, social and political decisions associated with affordable housing could be in tension, especially with limited funding.

“… people delivering … housing projects for the council find they’re told to do an awful lot of things and meet an awful lot of objectives but they’re not told which is important and when it comes down to it, it makes it really difficult for them to steer the path through actually the trade-offs between the different things that they’re delivering ‘cause everyone wants everything essentially” (BL-298, local government, sustainability)

Because of reductions in central government funding, and multiple short-term pressures, there was a feeling that “local authorities are now just completely dependent on their business rates and their council tax” (UB-468, local government, public health), therefore development was accepted to secure investment not available from internal budgets (as well as achieving housing targets).

“… central government’s funding of local authorities is going down, down, down, down, and resources are more and more stretched and there are savings targets that have to be met today, which always trump tomorrow because the implications of not meeting savings targets to balance the books today are significant, like huge for the organisation.” (RK-308, local government, property)

“… the more people you’ve got living in the city, the richer you are and particularly if they’re working and they’re paying their taxes so … that has to drive it [development] because how do we survive otherwise?” (GS-445, local government, elected member)

Local government agenda, led by politicians, could influence urban development. However, there was reluctance to take potentially controversial stances, particularly for challenging car dominant environments. Community engagement for active transport interventions was described as ‘activation’ for ‘promoting’ active neighbourhoods, with recognition that cultural change takes time.

“It’s politically easier for the councils to allocate a site miles away where everyone has to get around by car and cycling isn’t viable, public transport isn’t really viable either, which to me suggests that you know these councillors on these planning committees are more concerned of not losing local elections than they are with pursuing good urban development, policies that include benefits for health and wellbeing” (TS-495, national third sector organisation, urban planning and housing)

Tension was perceived between neighbouring councils with different priorities, with suggestions that councillors may not care about the health impacts of urban development for residents living outside their administrative area.

“… it was quite evident … when I went to that planning committee how little responsibility or care neighbouring local authority councillors have for urban [city] air quality and also the health of its residents. They could not care … They’re not elected to represent urban [city]. ‘So [city] are going to have the impact, it’s not our problem’ …” (GS-294 local government, transport)

Health was often not expressed as a priority but there was evidence of strong support for healthy place-making by local government officers. It just may not be framed directly as ‘health’, rather “It tends to be talked about in terms of carbon, air quality, congestion externalities rather than health.” (HI-491, regional government, environment and transport).

#### 3.1.3 Private sector priorities

Private sector priorities tended to focus on maximising profits, which could conflict with healthy urban development outcomes. However it appeared that some elements of the market (e.g. long-term and institutional investors) were increasingly needing to consider sustainability and wellbeing issues. Developers’ investors for real estate operate in global financial markets and seek “a reasonable risk-adjusted return” on investment (DL-152, private sector, real estate finance).

“… it is an industry that goes, ‘We’re functioning perfectly well, just making lots of money carrying on like this, so why would we change?’” (JB-199, private sector, property development and investment)

Seeking short term profit can reduce incentives to create healthy environments which may be more expensive. Prioritising profit may promote gaming behaviours such as land banking or flipping. Investors’ interest in health appeared mixed: some property developers expressed that “[investment bankers don’t] give a damn about place making or really they don’t give two hoots about people’s health. They want return on their investment” (MF-177, private sector, property development). However, various interviewees from global financial services companies highlighted that real estate investors, especially institutional investors such as pension funds, increasingly see sustainability and wellness as critical factors that affect their return on investment and consider them very much in their investment decisions in this shareholder society.

“All these companies, they need debt and the debt capital markets are tightening the screw on everything, so when your shareholders and your bond holders are asking you, ‘What are you doing about wellness and sustainability?’, it quickly jumps up the agenda!” (DL-152, private sector, real estate finance)

Land sales reportedly focused on financial value, not future land purposes, and interviewees discussed pressure to reduce design and build costs where developers paid too much for land due to competition.

“… sometimes they have purchased a site for perhaps, dare I say it, too much and that influences, A, the quantum of development they’re seeking to achieve to make a profit and B, the quality of design that you get at the end of it, because margins are so tight.” (WB-331, local government, urban development)

Developers, especially large, dominant house builders, were likely to have a well developed product, have “strong political voice” (GN-190, private sector, property investment) and may be perceived as low risk for investors. They were reportedly unlikely to want to change their business model and mixed use/tenure investments were described as more complicated, deterring investors.

Some developers were “in it for the long term” (TH-329, local government, urban planning) which may increase consideration of health and wellbeing issues.

“… where you’ve got an established developer who’s going to be there for the foreseeable future. There’s much more emphasis placed on not just flogging properties which obviously they all need to do, but the quality of the environment, and that all comes into play and that’s where you end up with the best results.” (GS-294, local government, transport)

Various private sector actors appeared motivated to improve people’s health but consultants could have limited scope to influence clients and some interviewees said it was difficult to find committed staff with perseverance to deliver good quality outcomes.

### 3.2 ‘Rules’ and relationships

Stakeholders’ priorities are influenced by formal control mechanisms for urban development decision-making, including policies and legislation (‘rules’), as well as by relationships between actors. We describe key ‘rules’ and relationships below to highlight important structural and relational issues that influence creation of healthy places.

#### 3.2.1 ‘Rules’: policies and legislation

Many centralised policies, regulations and laws were discussed that controlled urban development decision-making, alongside local planning policies and frameworks. However, these may be insufficient to enable healthy place-making due to focus on process over outcomes, power asymmetries, perverse financial incentives and lack of enforcement.

Some stakeholders discussed how ‘rules’ sought to control private sector developers who may otherwise produce poor quality developments, but there was criticism of focus on process rather than outcomes.

“… the property sector from which I come is untrustworthy basically. We’ll leg over communities and places in order to make a fast buck and, I’m sorry, that’s how it is. You know, it’s what happens and so therefore the planning system and any government support to get anything off and away is beset with safeguarding so there’s an awful lot of tick boxing that goes on” (LP-94, private sector, property development)

There was a view that people may cherry-pick arguments to oppose local development. An example was the motor lobby using inadequate consultation processes as reason to oppose restrictions on vehicle access in low traffic neighbourhoods.

Although local policies could be influential developers were said to “still argue” with local authorities (TH-329, local government, urban planning). The planning system was described as rigid and slow to change, which limited opportunities for innovative ways to incorporate health and it was difficult for local policies to be more ambitious than national policy. Legislation was described as much more useful than guidance.

“… legislation rather than guidance, that’s what we’re always told by developers when we try and push it. Because we’re always pushing too far because we want to get the best and they’ll say, ‘well on what basis are you asking for that, what policy? … that’s just guidance, you don’t have to do it’, and that’s the bit that annoys me the most and it’s only really through policy and legislation that ensures that …” (GS-294, local government, transport)

Urban development’s legal and regulatory mechanisms require specialist legal skills in a variety of legal domains (e.g. building regulations, environmental law, public sector duties, contract law, land law). It appeared there could be power asymmetries between some well-funded commercial developers, with easy access to quality legal advice, and many local authorities which lacked legal expertise, time, and resources. Due to the lack of resources, legal uncertainties, and disproportionate costs of appeal, planning officers may adopt risk-averse attitudes and follow ‘tick box’ approaches, rather than enabling quality place-making. Lack of enforcement was well-known, enabling private sector actors to maximise profits over compliance.

“… it takes a lot for a local authority to have the resource to fight a developer because you need a lot of money behind you to get lawyers involved and enforce it, and often they can’t afford to because they’re too busy paying for children’s social workers, quite rightly. So yeah, there’s a resource issue around enforcement, for sure.” (SC-335, local government, urban development)

Regulations that defined minimum standards were criticised because they were insufficient to ensure healthy urban development. They reportedly could exacerbate inequality as only wealthier places were able to negotiate higher standards.

“Unless you have national minimum standards enforceable through planning, the poorer places ended up with the worst quality developments and those are the places that already have the health problems because of the huge link between social and economic deprivation and health. … in wealthier areas the councils are able to push for better standards and get better standards but in poorer areas they’re not and so that’s one way in which the decision-making process is likely to exacerbate health in-equalities’ (AG-68, third sector, sustainability and health)

Some interviewees suggested that regulation, taxation or policy change was needed e.g. to price in health and environment costs upfront; or for finance and credit regulations to influence housing affordability and accountability to stakeholders. Levelling up differences in tax arrangements on home ownership and rental markets, as well as on VAT subsidies for new housing developments, so they aligned with retrofitting, was also suggested. However some thought that regulations were sufficient – they thought implementation of regulations were the problem.

Despite limitations of ‘rules’ to enable healthy development the law was perceived as an important tool to achieve policy objectives, including on politically challenging local issues. Landmark legal cases challenged national legislation and may be a catalyst for societal and legal change.

“… that case in London of the child who died of an asthmatic attack and how the mother’s taking it through the courts and I think … that’s a legal event that’s going to have a long-term change I think.” (DI-99, private sector, urban planning)

#### 3.2.2 Influential relationships

How the ‘rules’ for urban development were followed could be influenced by formal and informal relationships, with values and motivations affecting how environments associated with health were considered.

In England’s discretionary planning system negotiations occur between developers and local authorities. Interviewees said development costs were opaque and negotiations involved “brinkmanship” (SC-355, local government, urban development). Developers were said to “play local authorities off against each other” (QT-613, local government, transport) by threatening to develop in another local authority. An alternative to “the traditional slightly combative situation with developer/council” (MK-132, private sector, property developer/investor) were partnerships between developers and local government which could be beneficial where values aligned. However, one local government interviewee said lack of capacity could restrict such major procurement processes.

Developers appeared to have close connections with many stake-holders across the system. Some interviewees talked about the importance of working with people they know and trust. However, there were accusations of an ‘old boys network’ in some private sector organisations which may limit diversity of views, including lack of representation by young people, women and minority groups.

“… it’s a really biased industry. You need to be a white middle-aged man to be listened to. If you’re an immigrant, if you’re a female, if you’re young then you know nothing.” (CP-044, private sector, property development consultancy)

There was discussion that developers, or their consultants, may seek political support for development, and also seek to influence national and local policy-makers.

“[The MP] introduced us to the [Homes and Communities Agency]. He called all the right people at the council and all the doors that had been a crack, they all opened. Everything opened.” (MF-177, private sector, property development)

‘Relational capital’ was discussed as a way to initiate projects – people “that have really cared and have been really willing to drive it and eventually you bring everyone else” (KS-368, Local government, housing). Public health officers appeared able to influence non-health colleagues.

“There is that really strong critical challenge I think internally which is really really useful … [Public health] do challenge everybody to say why are we not doing this or why are we doing it this way and we mustn’t forget health when we’re developing policy and so on.” (XT-951, local government, transport planner)

#### 3.2.3 Limited community influence

Community consultation is required for major developments to ensure that public voices are heard. However, these tend to be insufficient to ensure creation of healthy places due to lack of representation, trust, awareness and funding. There was criticism that traditional consultation activities did not seek meaningful inputs, were not representative (more commonly older, more affluent, educated and male) and occurred too late to be influential. This was reportedly because of pressure to deliver development at pace and lack of trust by developers towards engaging communities - some felt that community consultation could be a threat to financial viability because of potential costly delays caused by public opposition. However, others suggested meaningful engagement could speed up gaining planning permission by removing public objections.

“… there’s such a rush for development at the moment that sometimes – and they don’t want to wait to listen to the community” (TF-285, local government, other)

“… all of these familiar commercial pressures which will work directly against what you fear local people will, quite rightly, demand. There’s both an unjustified and an entirely justified fear of engaging local people more.” (AS-192, third sector, property finance)

Lack of public trust and knowledge about the complicated planning system was described as a barrier to engagement. One interviewee said “people think planning has got so much power” but “most of the time we can’t get everything and we have to make compromises.” (WB-331, local government, urban development).

“… [communities] instantly don’t trust you. They don’t. Will not let you in because we don’t trust you, ‘we don’t know what you’re gonna do to us’ … if it’s a developer they just think the developer’s here to make money. If it’s the council they sort of think, well the council’s going to impose something on us and then they’re going to break some promises as well.” (JM-313, local government, housing)

Educating the public was suggested as needed by some interviewees. While new residents may engage in social media to critique the quality of development, which may drive quality, there were also reports of volume housebuilders using Non-Disclosure Agreements to keep occupants quiet about poor housing. Lack of time and awareness to engage, particularly in more deprived areas, was reportedly a problem. Interviewees said this could result in poorer quality development due to reduced oversight.

“The truth is in [poorer ward] there is absolute apathy, to be honest with you. … the general public in [poorer ward] have just got better things to do …. but meanwhile in [affluent wards] … they’re jumping up and down, writing into full council, putting in questions to cabinet, or making an absolute stink … the silent voice doesn’t mean the issues are not there and actually, sometimes it’s more systemic”. (PJ-385, local government, elected member)

Although there appeared difficulties in accessing engagement funding local government stakeholders appeared enthusiastic to engage communities, describing learning from the public as “understanding customer intelligence” (RK-308, local government, property development).

“… we won’t visit a site as much as somebody who actually lives there and experiences it every day. So I kind of see local stakeholders as – we need to see them as allies because at the end of the day we both want the local environment to improve. And they’re our eyes and ears if you like, sort of local geographers on which we rely on” (GS-294, local government, transport)

### 3.3 Justifying a focus on health

Many stakeholders did not seem to view health as their responsibility. While public health was acknowledged as important across government there was no recognised champion to promote health mainstreaming beyond health departments. We describe how clearer definitions, evidence and resourcing for healthy places may be helpful.

#### 3.3.1 Defining and evidencing health

Clarifying what was meant by ‘healthy’ development, using evidence, could increase objectivity for decision-making to prioritise health. Quantitative measures, including framing health in commercial terms, may incentivise some stakeholders.

Health and wellbeing outcomes can be associated with a broad array of environmental factors. Defining ‘healthy’ development appeared a necessary, albeit often missing, step to clarifying how to improve health outcomes. The interview questions did not define ‘healthy’ and some interviewees assumed narrow definitions (i.e. clinical, rather than prevention), whereas others described health as “too broad … because it embraces so many different elements” (GW-402, Local government, housing). Proxies for health, such as environmental conditions, active travel and air quality were described by some interviewees.

There was criticism some health bodies inadequately focussed on wider determinants of health and suggestions that national government did not recognise the associations between urban development and health.

“… if we go to the very most senior level of government and economic level, I think this country struggles to recognise the relationship between place, development and health outcomes” (BX-596, regional government, transport)

The lack of clarity about ‘healthy’ development appeared to make it difficult to consider trade-offs e.g. between environmental and health outcomes. The lack of clear epidemiological evidence demonstrating associations between the built environment and health outcomes appeared to limit ability to object to planning proposals. This could result in accusations that comments about health impacts on planning applications were “off-hand, unsubstantiated” (HM-336, local government, urban development). It was thought that health could not be used to object to planning applications because it would not stand up to examination by the government’s Planning Inspectorate. Greater objectivity appeared necessary.

“… you’ve got this horrendous policy in the [National Planning Policy Framework] which says we can only refuse things if the impact is severe. Now define severe, you know, I was asked this in a public inquiry … people living in high buildings, mental health issues associated with that, isolation, that’s very hard to quantify.” (GS-294 local government, transport)

Ambiguity about ‘healthy’ urban development appeared to result in individual interpretations, which gave scope for ideology, personal preference, or unsubstantiated claims around health.

“… they’ll be some in society who think a healthy place is basically an open meadow and there are others that think it’s a well-designed pair of houses, there are different definitions … the lack of a common view as to what it looks like allows people to kind of come up with their own interpretations. At which point they can just build a 5,000-home housing estate and one small play park in the middle of it and say ‘look it’s healthy’”. (TH-414, national government, policy)

Lack of follow-up post occupancy evaluation appeared to result in limited understanding about residents’ views of supposedly ‘high quality’ developments. Monitoring and evaluation of major government investments also tended to be limited, including long term evaluation of transport investments which were described as hiding induced demand.

“Very rarely … does central government ever check up on what its money’s been spent on … they expect to see the results on the ground so they don’t need to check on what they’re spending, if you see what I mean” (DW-529, national government, civil servant)

Framing health in commercial terms may appeal to developers, particularly if they increased house prices. Some voluntary certifications were said to increase rental yields by attracting occupiers (e.g. BREEAM or WELL certification). However, it was also suggested that “you can cherry-pick which [standard] you want to use” (JB-199, private sector, investor/property developer) and therefore it was easy to game.

A quantitative measure of health, similar to carbon metrics was described as potentially “hugely helpful” (NB-134, private sector, property finance) and monetising health benefits was seen as useful by some, although currently “sometimes it’s just finger in the air stuff” (TH-414, national government, civil servant). Some said real estate stake-holders and politicians often lacked evidence-based decision-making, instead using “experience and gut feel” or “a political view” respectively (PR-140, private sector, property).

Where evidence was available interviewees discussed “choosing the right language and the right argument for who you’re trying to talk to” (AK-247, national level public sector, property development), which can involve both qualitative case studies and quantitative data.

#### 3.3.2 Obtaining resources to focus on health

To enable healthier places interviewees suggested funding priorities needed to shift, particularly by national government since local government resources were limited, bidding for funding could be inefficient, and developers likely unwilling to pay additional costs. Demand may affect the market’s willingness to fund these costs.

Some interviewees thought national government should provide additional funding to local authorities, for example via subsidies for affordable housing, especially in poorer areas with high housing needs. However, long-term population benefits, compared to short term costs, did not appear to be incentivised in the current short-term political system.

“Politicians want sustainability and to spend less money on things, but then don’t actually invest in prevention and the things that will lead to that. Housing being one of them that could save NHS money”. (AH-240, national government, health).

Local authorities could bid for additional national funding, however there was criticism that there were limited opportunities to consider health in funding bids. Bidding for ad-hoc pots of money was thought to be ineffective for long term, sustainable and healthy investment in urban development and could increase inequality where councils with fewer resources may not have capacity to bid.

“If you’re also bidding for things that’s resource intensive. … maybe that’s not the most efficient way, because if you’re always bidding for ad-hoc individual funding streams you might be successful, you might not. You have to pick yourself up and dust yourself down and think about how you reframe those bids. It takes effort.” (EC-300, regional government, urban development)

Although described as difficult, health economic evidence may help persuade Treasury of the value for money of investing in healthy urban development. Interviewees said this was important to influence other government departments to focus on this agenda.

“I think the Treasury would probably focus too much on the costs as opposed to the longer-term benefits of being able to measure those. I think instinctively it feels like better being able to show that, to sort of tell that story and share that thread and show how in different places these investments have made this difference and over time it has saved this, and over time it has resulted in this” (JH-388, national government, property finance).

However, influence of health economic evidence appeared limited where costs were borne by developers or different parts of government, and benefits felt by residents. One interviewee pointed to huge budgets in transport sectors, with health economic benefits described as a “*drop in the ocean compared to the amount that they’re already spending year on year” (*DC-93, private sector, transport).

Some public sector interviewees expressed hope that the Covid legacy may shift demand for healthier urban development, particularly relating to better access to greenspace. Therefore developers would “follow the money” (SC-335, local government, urban development) and provide better places. However, house prices were perceived as more influential than features associated with health for many buyers because of high prices.

## 4 Discussion

### 4.1 Main findings of this study

**Fig. 2:**
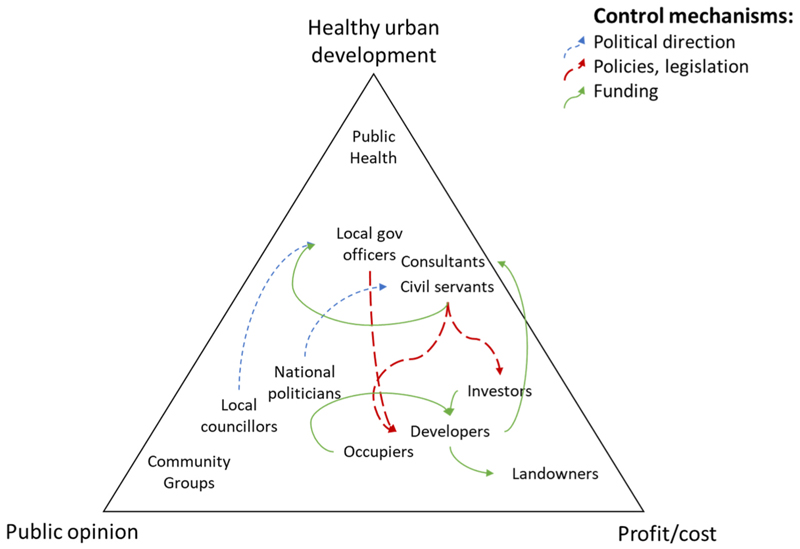
Conceptual model of dominant priorities and control mechanisms for some stakeholders involved in decision-making for urban development.

The complex system of urban development decision-making is composed of many types of stakeholders across sectors and organisations. Stakeholders recognised complexity of the system and we identified differing and often competing priorities, with vested interests and inbuilt inertia, making it difficult to prioritise health and wellbeing outcomes in urban development. We summarise aspects of the system in Fig. 2 to highlight three potentially conflicting priorities: healthy urban development, profit/cost and public opinion. This situates dominant stakeholder priorities amongst these key variables (closer to the corners equates to likely greater priority).

The arrows in Fig. 2 highlight our interpretation of key control mechanisms across stakeholders: policies/legislation (‘rules’), and funding. Political control is also shown to demonstrate relationships, and likely differing priorities, between political and non-political actors in local and national government. The figure highlights that public health and community groups are not clearly connected to these important control mechanisms, suggesting that they have low levels of influence in the system.

This diagram is a simplification and in some cases the gap between healthy urban development and profit/cost could be small, such as where healthier places drive higher returns on investment. Similarly, the gap between healthy urban development and public opinion could narrow if people demand healthier places. We hypothesis that justifying a focus on health outcomes through clearer evidence and objective measures of healthy development could help bring the corners of the triangle together as priorities align, rather than compete.

**Fig. 3:**
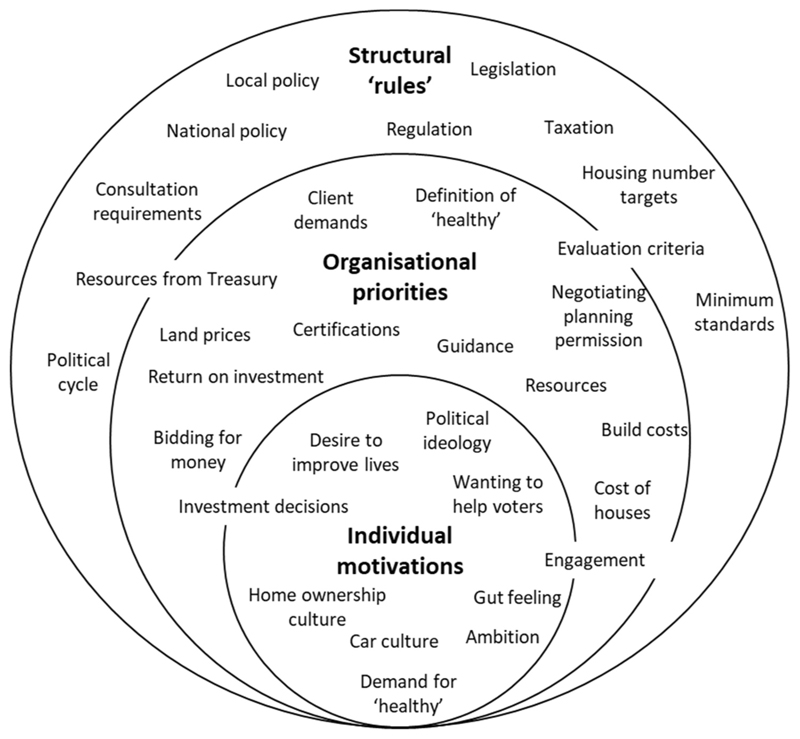
Summary of issues at different levels that appear to affect urban development decision-making and population health outcomes.

We now consider the broad system of urban development decision-making through the lens of individual actors, organisations, and structures. This builds on the socio-ecological model (Bronfenbrenner, 1978) to consider how health considerations may be better integrated, considering the importance of ‘rules’ and relationships, and the challenges of competing priorities across stakeholders. This framework is useful to identify how multiple parts of the system can influence outcomes, while keeping awareness that parts of the system affect, and are affected by, multiple other parts (Hawe et al., 2009). It demonstrates that effectively integrating health into urban development decision-making does not rely on a sole level in the system - interactions between individual motivations, organisational priorities, and structural rules are important. Focusing on any one of these levels is likely to be insufficient to bring about meaningful change and multiple interventions targeting these different points in the system are needed (Atkinson et al., 2015; Rutter et al., 2017; Sallis and Owen, 2015). Fig. 3 presents a summary of issues identified in our study at these three levels (note some span multiple levels). These range from more subjective issues relevant for individual actors, to more objective, structural issues across the system. Incorporating our findings about the importance of defining and evidencing health in urban development we suggest that greater clarity concerning associations between environmental features and health is important across levels to increase demand for healthier place-making; enhance health as an organisational priority; and integrate health within the ‘rules’. These are discussed in more detail below.

### 4.2 Findings in context

#### 4.2.1 Individual actors to demand healthier places?

Individual level behaviour change interventions could affect motivation for healthier decision-making, but there may be unintended consequences (Hawe et al., 2009) since, within existing organisational and structural contexts, demand for healthy places may influence supply which could affect affordability and exacerbate inequalities.

Members of the public, as customers of developers, may affect the market and it appeared developers may increasingly consider feedback from residents, such as via social media. While some occupiers/residents may demand healthier environments (perhaps in part due a legacy from Covid-19) others may not if financial issues, especially affordability, are more significant. Higher prices due to ‘green premiums’ were discussed, but without consideration of affordability, which may exacerbate inequalities (Browning et al., 2022). Increased demand for healthier places could result in a ‘health premium’, although there is mixed evidence of objective measures of health influencing house prices, such as air quality or noise (McCord et al., 2018; Pinchbeck et al., 2021) suggesting perception of health may increase prices.

Emotions and values are known to influence decision-making (Knaggård, 2015; Cairney and Kwiatkowski, 2017; Cairney and Oliver, 2017). This could be used to motivate influential actors to support healthier place-making, such as designers and planning committee members. However, these actors may still be limited by structural factors. The case of Ella Adoo-Kissi-Debrah, whose death was associated with air pollution from road traffic in London (Dyer, 2020), was discussed by multiple stakeholders and such tragic examples could help contribute to a shift in narrative about environments and health. Such examples could also promote issues of ‘fairness’, as suggested in ‘health in all policies’ guidance (Local Government Association, 2016).

As a ‘tangled’ problem (Dawes et al., 2009) the public may need help to understand how to influence development within existing ‘rules’ (e.g. the financial and legislative systems in which developers and governments operate) and build trust. Greater public understanding about environments and health and less individual subjectivity of what makes environments healthy may help facilitate public demand for genuinely healthier placemaking, and reduce negative outcomes, such as low-density development exacerbating reliance on private cars. However, the UK is highly individualistic (Hofstede Insights, 2022) and the narrative around health often focuses on individual responsibility over structural factors (Department of Health and Social Care, 2018; McCormack et al., 2020), despite evidence that health interventions requiring high individual agency are less likely to be effective or equitable compared to structural changes (Adams et al., 2016). Targeted interventions could address political ideology that values individualism over collectivism to influence elected representatives’ willingness to support environmental change, particularly where there are public concerns, such as about restricting car use.

#### 4.2.2 Organisational decision-making to enable healthier development?

Across the system organisations have differing priorities and objectives (Blackman, 2006; Finka and Kluvánková, 2015; Holmes et al., 2017). To influence these greater clarity and objective definitions about healthy environmental features appeared necessary to justify this focus, particularly as health tends to be lower priority than other organisational aims, such as profit or addressing social needs. Interventions could include development of objective definitions and measures for health to be considered within evaluation criteria for investment covenants, loans, procurement contracts and grants. Increasing recognition of relevant environmental features, beyond simply activity or housing type (McCormack et al., 2021) may be needed. This could involve more evaluation of natural experiments and prospective cohort studies to build specific evidence bases, including for how different demographics interact with different environments to develop ‘believable stories’ (Le Gouais et al., 2021). However, stakeholders appeared to need support to understand epidemiological evidence, such as how confounding may be accounted for. Considering functions of interventions, rather than context-specific effects, could assist with transferability (Ogilvie et al., 2020a) but if this translates to voluntary guidance it may not be impactful.

Understanding influences across the complex system may challenge beliefs of some investors and developers who thought they had a limited role in shaping health outcomes. Increasing knowledge about healthy urban development could also help influence alignment of organisational goals to support collaborative working, although consideration of informal interactions and negotiations, not only formal processes, are needed, including (as for any intervention) potential unintended consequences (Alhassan, 2021).

Clarifying benefits of meaningful public involvement to organisations could also be done, involving ‘top-down’ ‘activation’ for promoting active neighbourhoods, or ‘bottom-up’ knowledge gathering (Buse et al., 2012) as risk mitigation for organisations to increase community support for change (Brill, 2019). Tackling the ‘old boys network’ described by some private sector actors to reduce prejudice towards engaging with diverse publics may also drive positive change.

Funding and cost/profit issues dominate decision-making for organisations. Although there appeared some shift in investor demand to support more sustainable projects, if healthier development is more expensive new funding mechanisms may be required (an example of additional challenges following shift in individual motivations towards healthier development (Hawe et al., 2009)). Some interviewees thought government subsidy was justified, particularly if long-term savings were considered, which may be aided by health economic modelling. However, costs and benefits fall on different organisations, therefore investing in healthier places is not adequately incentivised, and impacts on wider government budgets are unclear.

The dominant home ownership culture in the UK may facilitate short-termism by house builders to prioritise sales over long term health and sustainability. Changes to business models to promote long term stewardship, such as ‘Build to Rent’ (British Property Federation, 2017), may help. Modern methods of construction (MMC) are also suggested as a way to build with lower costs (Davies, 2018). However, there may be unintended consequences with these relatively novel approaches.

In local government organisations, limited funding and political pressures can result in ‘tangled’ problems (Dawes et al., 2009) associated with issues of governance, capacity, trust and ambiguity in responsibilities. Interaction of public bureaucracies across social, cultural, economic and political contexts results in additional challenges of working in a complicated, as well as complex, system (Vogler, 2019). It suggests that while public sector knowledge networks may be beneficial (as advocated by Dawes et al. (Dawes et al., 2009)) broader structural issues must also be tackled to address the challenges of creating healthy environments.

#### 4.2.3 Structural change to integrate health in urban development?

While motivations of individual actors and organisational priorities may affect some decision-making for healthy urban development, structural ‘rules’ set out by other organisations may be most influential, particularly policies, legislation and funding decisions. This includes from central government, such as housing targets and tax regulations that influence decision-making. However currently health is often not part of the ‘rules’. While this suggests a prime intervention target area, policy change itself is highly complex and can be slow, requiring ‘windows of opportunity’ (Weiss, 1979; Kingdon, 2010; Cairney, 2020), alongside changes to values and motivations of actors in the system.

Even where ‘rules’ do support healthy development they may not be followed, particularly when loosely defined (UWE Bristol, 2021). While national policy promotes healthy communities (Minstry of Housing Communities and Local Government, 2021) previous research has found inconsistencies and lack of knowledge about health impacts by the Planning Inspectorate when cases go to appeal (O’Malley et al., 2020). This suggests that organisational interventions involving greater objective evidence about health impacts could support interpretation of ‘rules’ for healthier places.

Interviewees suggested financial regulation and taxation changes could promote healthier places. These approaches have successfully influenced private sector organisations associated with other determinants of health e.g. sugar sweetened beverages (Alvarado et al., 2019; Pell et al., 2021). Enabling such structural changes will likely require multi-level approaches to also influence individual actors and organisations.

### 4.3 Strengths and limitations

The views of many different stakeholders are included in this study, from across the urban development system, although this included more property development stakeholders than transport stakeholders. Not all the original sample participated and we used some snowball sampling which may have influenced responses received.

As an exploratory study ALG conducted a thematic analysis. She conducted 16 of the local government interviews, with colleagues from other disciplines conducting other sets of interviews and summarising their findings (Bates et al., 2023). Recognising the active role of the researcher (Braun and Clarke, 2019) the findings presented could have differed if conducted by other researchers.

The large data set meant it was not possible to explore all findings in depth in this paper and some issues are explored in other papers e.g. (Montel, 2023).

Our study was conducted in England, a high income country (with areas of socio-economic and associated health disparities) with a discretionary planning system. Stakeholders had experience of different contexts but some insights may not be transferable.

## 5 Conclusions

The urban development system is complex, with multiple stake-holders with differing priorities and goals. The drivers of individual actors and organisational priorities affect motivation and ability to integrate health, while structural factors, including policies and legislation (‘rules’), can also be influential in the complex system. These issues can influence one another: behaviour change interventions targeting individual actors may in turn affect some organisational priorities, which could also go on to influence structural factors. However, a multi-faceted approach is required for change at all levels in this complex system and interventions to better integrate health in urban development decision-making should target multiple levels: individual motivations; organisational priorities; and structural ‘rules’. It is the combined effect of interventions at different points in the system that can lead to meaningful and enduring changes. This may require greater clarity and consensus around what makes urban development ‘healthy’ to increase demand for healthier places, justify its focus as an organisational priority, and to integrate it within structural ‘rules’ to facilitate healthy place-making.

## Figures and Tables

**Table 1:** Summary of interview participants.

Stakeholder role	Local/ Regional government	National government	Private sector	Other	Total
Property development	5	2	24	–	**31**
Urban planning	15	3	5	3	**26**
Finance	–	3	18	–	**21**
Transport	6	3	3	1	**13**
Public health	7	2		2	**11**
Politician	8	1	–	–	**9**
Environment/Sustainability	3	2	1	1	**7**
Other	5	4	2	3	**14**
**Total**	**49**	**20**	**53**	**10**	**132**

## Data Availability

Data will be made available on request.
